# Monthly Summary

**Published:** 1886-06

**Authors:** 


### Monthly Summary.

SOMETHING NEW IN MAKING RUBBER PLATES.—One of the features introduced as a novelty, at the late meeting of the Mad River Valley Dental Society, was a method of making vulcanite plates without the teeth attached; in other words, vulcanizing the rubber on the plaster model, using the plate or plates thus obtained instead of a wax base-plate for securing a bite, and finally attaching the teeth by a second process of vulcanizing. The idea is original, we believe, with Dr. Bradley, of Dayton, O. The advantages claimed for the method are such as apply to swaged metallic plates, viz.: greater accuracy in securing a "bite," in difficult cases, the opportunity of determining at an early stage of the operation whether a good fit has been secured, also the advantage of allowing the patient to wear and accustom himself to the plate before the teeth are attached. Dr. Bradley thinks the method of special advantage in all partial lower cases. The narrow rubber band at the lingual base of lower anterior teeth may be strengthened by cutting a groove with a fissue or wheel-bur, fitting into this a *brass* wire, and vulcanizing rubber over it. For attaching the teeth, the surface of the plate is treated with a solution of persulphide of carbon, after which, the teeth being in position, more rubber is applied, and the piece again vulcanized.

Dr. Adams, of Dayton, stated to the Society that the packing of the rubber, when the plate is first made may be done as ordinarily, with the fingers and a blunt burnisher, after which the surface is covered with tin foil, the case invested and vulcanized. He dispenses with the wax base-plate, and consequently with the various steps of varnishing and oiling, and a second pouring of plaster in the flask. To be more explicit, the rubber strips are packed upon the face of the model *before* flasking at all, and then the model is invested at one operation and immediately vulcanized. It will be seen from this, that no pressure is brought to bear upon the rubber in closing the flask. Plates made in this way were exhibited to the Society. They appeared to be quite as hard, tough, and strong as any made in the usual way, giving no evidence of porosity.

A member stated that Dr. Haskell vulcanizes only thirty minutes, being careful that the flasks are steam-tight and kept covered with water.

Dr. A. Berry said he had not succeeded in the attempt to vulcanize in that time. He also stated that rubber packed without forcible pressure makes a stronger plate than when the rubber is powerfully compressed in the flask.—*Cinn. Med. and Dental Journal.*

---

CARIES OF LEFT SIDE INFERIOR MAXILLA.—Dr. B. H. Catching, D. D. S., before the Georgia Dental Society, said: This case, a very serious one, may prove of interest to the profession. A Sister of Mercy was suffering, as she supposed, from odontalgia in a lower left molar. She visited a dentist, who extracted, as he supposed, the offender, without giving relief. She returned to the same dentist, a short time afterwards, complaining of jaw-ache. Another molar was removed, without affording relief. A few months after, intense suffering, causing complete prostration, ensued. An abscess about the size of a tea-cup, formed at the angle of the jaw, involving the submaxillary gland of that side. This produced occlusion of the jaws.

Dr. F. H. O'Brien, an eminent physician, was called. The trouble, he saw at a glance, was a very serious one. He lanced the abscess, evacuating about a tea-cup of pus, so offensive that no one could remain in the room until it was deodorized. He became satisfied that it was a case requiring the care of a dental surgeon, and called the writer in. He had previously told the Sister that in all probability a part of her jaw would have to be removed. When I first visited her, in company with

Dr. O'Brien, I found her in a very despondent mood, with hardly enough vitality to take interest in our visit. Pyæmia was evidently manifesting itself. Her jaws were so occluded that I could not make an oral examination. The abscess was ordered washed out repeatedly by injecting with carbolated water, and instructions given that she endeavor to move her jaws frequently during the next twenty-four hours. On the next day I still found the occlusion so great as to prevent an examination. She was urged to repeated trials of separation. After the fifth day I could introduce my finger, with which I discovered a molar, very loose, remaining. With some difficulty it was removed, when with a probe the honeycomb surface was revealed, showing beyond doubt, in connection with the nature of the discharge, caries of the bone.

A few days later, when I had better access to the parts, and when the patient was able to undergo the operation, I laid the gum open from the ramus to the second bicuspid, and pressed it away on either side, which was easily done, as the adhesion to the bone was very imperfect. With a good sized scraper the diseased surface of the bone was removed. Aromatic sulphuric acid diluted was applied on cotton to the whole diseased surface. While I was treating locally, Dr. O'Brien was successfully treating systemically. After about three months, she was entirely cured.—*Southern Dental Journal.*

---

ARSENIC IN KILLING PULPS OF TEETH.—Arsenic will seldom produce pain if applied without morphia, and with tannin. Even if the tooth is aching at the time of its application, it will generally soothe it to death. It is sometimes desirable to precede its application with a little cotton saturated with chloroform. Morphia is an extreme irritant to a raw surface—try it on a wound or burn—and therefore instead of abating toothache caused by an exposed pulp, it increases it. The cause of pain in such a pulp is inflammation, which is not an increased flow of blood to a part, as generally supposed, but a clogging of the venous blood of a part, so that the blood cannot return through the veins with the normal freedom it is brought to the part. Tannin constringes the pulp, so that instead of the nerves being pressed by the swelling of the mass within confining walls, the whole becomes tanned and shrunken. Pain everywhere in the body is caused by pressure on the nerves, especially on their termini.

A good combination of the arsenic is: One part by weight of arsenic and two of tannin, to be made into a thin paste by one part oil of cloves and two parts creosote. The finer the arsenic is pulverized the less is required to do the work; in fact, but an extremely small amount of the paste should be applied. Twenty-four hours is generally sufficient to devitalize the pulp; though when this very small amount is used there is little danger in its remaining longer, and generally the pulp will be found sufficiently tanned to be brought away whole. Sometimes the paste has to be applied a second time. After touching the pulp with this paste loosely, filling the cavity with cotton, and then on this drop a little sandarac varnish. Any filling which necessitates pressure on the pulp will, of course, produce pain.—*Items of Interest.*

---

TUBERCULOSIS OF THE BUCCAL MUCOUS MEMBRANE.—There are four different ulcerations which may attack the buccal mucous membrane. All four are of a grave nature, but one, if early recognized and correctly treated, may be cured, provided it has not yet impregnated the whole system. These four are carcinoma, lupus, syphilis, and tuberculosis. Mr. David Hauseman, of Kiel (*Virchow's Arch.*, 103 bd., 2 heft, 1886), has recently drawn our attention to these ulcerations, and published the result of his investigations.

According to him, the main point, the correct diagnosis, can only be made by excision of the new growth and later examination of the same under the microscope. For this purpose, while the presence of giant cells with granulation tissue might answer in a general way, the discovery of the tubercle bacilli forms alone the definite proof, but of late it has been made so easy to detect their presence that almost a tyro in the art, provided his microscope be powerful enough, can demonstrate their existence. The tubercular nature proved, a thorough excision will establish a radical cure, provided the system is not yet affected, *i. e.*, provided there is no dullness over the apex of one lung.

Lupus can generally be excluded by noticing for one or two weeks the effect of specific treatment, as under the latter any syphilitic sore would at once improve. While the diagnosis between cancer and lupus is easy, that between the latter and tuberculosis is more difficult, as the bacillus of lupus and that of tubercle greatly resemble each other. Here the pure-culture, or at least the color-test will decide.

The advantages to be obtained from following H's advice is clear, as an early excision of the tubercular growth will prevent a return and a general infection, while of the other malignant growths such cannot be said with the same certainty.—*Med. and Surg. Reporter.*

---

INTIMACY BREEDS CONTEMPT.—The relation of dentist and patient—as it is with physician and patient—is so close that nothing but a dignified reserve can prevent an intimacy which breeds contempt. Because patients come to us for professional work is no reason we should expect to become their friend and acquaintance and yet many dentists, before they have completed their professional engagements, seem to think they must pry into all the private affairs of their patients—their failures and successes in business, their likes and dislikes in society, and even their more private concerns. If their patient is of the gentler sex, this intimacy too often includes a maudlin sentimentality and repulsive improprieties. It is astonishing such dentists are tolerated with in any society. They certainly lose the respect of their best patrons. Intimacy breeds contempt.—*Items of Interest.*

---

A REMARKABLE CASE.—Mrs. F. A. Lewis died at her residence in this place on Friday morning last, aged about sixty years. Mrs. Lewis for many months past has suffered intensely from an affliction which completely baffled the skill of the physicians of this city, who have assiduously waited upon her, as well as physicians elsewhere, whom she had consulted. Such was the singular character of the malady that no surgical operation suggested itself for her relief. Some days before her death she asked that a post-mortem examination should be made in the interest of science, and that afternoon the same was made by Dr. Martin, assisted by Drs. Roy and Ashton, when a tuft of hair was discovered inside of her as large as a man's fist. The physicians say that no such case is recorded in the medical works, and has not occured in their experience. Mrs. Lewis has resided in this place almost continously since her return from California, in 1851, when she was a passenger on the ill-fated steamer Central America, which was lost at sea, when her gallant captain, Wm. Lewis Herndon, a native of this city, and a number of the crew and passengers went down with the ship. Mrs. Lewis, upon leaving the ship, received from Capt. Herndon his watch, with the request that if saved she would deliver the same to Mrs. Herndon, then residing in New York city, which trust was faithfully fulfilled.—*Fredericksburg* (*Va*) *News.*

---

THE TREATMENT OF SICK HEADACHE.—Dr. W. Gill Wylie (*N. Y. Med. Jour.*) of New York, has produced excellent results with the following treatment: So soon as the first pain is felt, the patient is to take a pill or capsule containing one grain of inspissated ox gall and one drop of oil of gaultheria, every hour until relief is felt, or until six have been taken. Dr. Wylie states that sick headache, as such, is almost invariably cut short by this plan, although some pain of a neuralgic character remains in a few cases.

